# Assessing health service satisfaction among users with substance use disorders within the municipalities in Norway

**DOI:** 10.1186/s13011-019-0207-4

**Published:** 2019-05-06

**Authors:** Marianne Stallvik, Grete Flemmen, Jo Arild Salthammer, Trond Nordfjærn

**Affiliations:** 1 Department of Research and Development, Clinic of Substance Use and Addiction Medicine, St. Olavs University Hospital, Trondheim, Norway; 2 Center of Drug and Alcohol expertise, Clinic of Substance Use and Addiction Medicine, St. Olavs University Hospital, Trondheim, Norway; 3 Department of Psychology, Norwegian University of Science and Technology (NTNU), Trondheim, Norway

**Keywords:** SUD, Health services, Municipalities, Co-occurring disorders, Treatment, Psychiatric symptoms, Service satisfaction, User experiences

## Abstract

**Background:**

The purpose of this study was to assess what is associated with health service satisfaction among adults with a substance use disorder receiving services provided within different municipalities in Norway. An additional aim was to examine demographic and municipality characteristics, mental health, and types of substance use associated with health service satisfaction.

**Method:**

A cross-sectional partial explorative study was executed in 2017 among 491 service users with substance use disorders from 20 randomly selected municipalities. The sample consisted of 70% males. The sample majority were single and unemployed, and their main sources of healthcare were the general practitioner (78%), The Norwegian Labor and Welfare Administration (72%), and addiction counsellors (62%).

**Results:**

Overall satisfaction was negatively associated with age, size of municipality, Global Severity Index (GSI) and illicit substance use during the last 12 months. Satisfaction with practical help, such as housing, economy, work and education, was negatively associated with GSI and positively associated with onset of first alcohol intoxication. Satisfaction with personnel was positively associated with onset of first alcohol intoxication and negatively associated with municipality size, GSI and illicit use the last 12 months. The results showed that more than half of the respondents (54%) to a large or great extent were satisfied with the overall services provided. The services they were less satisfied with were related to housing, economy, getting started with exercise and establishing a social network.

**Conclusion:**

The results show areas associated with satisfaction and domains where the municipalities can improve their services to meet the users’ needs and increase service satisfaction.

**Electronic supplementary material:**

The online version of this article (10.1186/s13011-019-0207-4) contains supplementary material, which is available to authorized users.

## Background

Investigating user satisfaction within health services is of high importance and is increasingly recognised as an indicator of service quality since satisfaction may be a feasible indicator of whether patients’ needs are properly met [5, 27, 50]. Such evaluation can highlight aspects of care that need improvement and give an idea of future service needs. Understanding user perspectives on treatment has been shown to be important in improving health care services [14, 48] and user satisfaction is an important supplement to other quality indicators like abstinence and treatment retention for evaluating substance use disorder (SUD) services [47]. Instead of focusing solely on patient characteristics when considering the quality of services for people with SUD, it has been argued that research should shift the attention to user satisfaction with staff and other treatment factors [27]. A recent review even concluded that further research on demographic data is of limited value [8], and they recommend studying what type of services are suitable to meet service users’ needs and their satisfaction with the services.

Higher levels of satisfaction has been linked to retention in health services, and an association between low health and reduced service satisfaction as well as increased risk of dropout has been identified in several studies [26, 29, 31, 39, 40]. The degree of satisfaction has been found to be related to higher treatment compliance, increased involvement in treatment and with users taking more advantage of the services provided [22, 49]. Satisfied patients are also more likely to stay in treatment, which in turn facilitate better treatment outcomes [11].

To our knowledge, there are to date no comparable studies on satisfaction with SUD services provided by the municipalities of this scale. However, several studies have been conducted in the specialized treatment services, and a majority of users use services from both specialized healthcare and the municipalities. It is also important to broaden research to include users of SUD services from the municipalities, since the municipalities are one of the largest providers of services for this group in Norway. The municipalities operate under different regulations and have other resources than the specialized clinics, and this might affect user satisfaction. Municipality programmes and treatment clinics differ in continuity of care, treatment duration and the extent that they help with housing, economic issues and social relationships [6, 16, 18, 23].

The specialised services have more trained personnel, higher degree of monitoring and more hospital services so they can handle more severe cases of the disorder. This also includes specialised services like detoxification and programs to handle symptoms related to mental and somatic issues. Services in the municipalities have directed their attention on social issues like housing, economy, daily activities, stabilizing the substance use and/or focus on prevention; issues that need to be handled for recovery from the disorder and to reintegrate people back to the society. There could also be less severe problems among service user in the municipalities, and this could contribute to differences in which factors that affect satisfaction among municipality service users and users of specialised treatment facilities. McCallum et al. [28] found more satisfaction among community care programs compared to hospitalized patients. Areas showing less satisfaction included staff competences, treatment access, and that their mental health needs were not met. A review of patients with SUD and co-occurring psychiatric disorders showed that they had low satisfaction in the same areas found in the previously mentioned study. Their dissatisfaction was not related to demographics or symptom severity, but tended to relate to treatment processes and outcome variables [39].

A high professional competence, ability and professional ethics are also related to increased user satisfaction, and this perceived competence among personnel as experienced by the users has a significant impact on variations in user satisfaction [9, 46]. Results from a recent study suggest that confidence in personnel and user involvement in treatment are connected to patient-experienced improvements in stabilizing their SUD problems and their perceived benefits from treatment [1]. Studies of satisfaction among inpatient users diagnosed with a SUD within the specialized health care in Norway showed that patients are most satisfied with professionalism and ethics among the personnel [19]. A study conducted at four mental health clinics in Canada found that continuity of care, having a case manager, and receiving help when needed was positively related to satisfaction, and number of needs among the users was negatively associated with satisfaction [17].

High overall satisfaction has been found in many of these previously mentioned treatment studies, but these overall measures of satisfaction might disguise areas where service users are less satisfied. Hence, the concept of satisfaction should be broadened to include measures of accessibility, personnel experiences, and services needed. Further, the lack of standardized satisfaction measures within this field makes it difficult to compare studies, and calls for further investigation of what differentiates satisfaction across different populations and settings. To our knowledge, no satisfaction studies have been conducted neither among users of community-based SUD services in Norway nor internationally with multiple centers and municipalities across the nation.

As previously mentioned studies have shown that factors related to satisfaction seem to be strongly related to personnel competence and treatment access [9, 19, 28, 46] In addition to personnel factors, the mental health of service users seems to directly affect their satisfaction [28, 39]. Studying satisfaction among users within the municipalities should include these variables, in addition to municipality size which entails the resource difference which might affect satisfaction. There is an ongoing shift in Norway towards integrating more SUD services in the municipalities rather than increasing services within the specialized health care system so it is important to increase knowledge and satisfaction on services provided within them. The core aim of the current study was to assess what is associated with satisfaction and services for users with Substance use disorders within the municipalities of Norway in terms of demographic, clinical and service-related factors. In addition we report the degree of satisfaction with the services provided. The study results extend the knowledge regarding service quality in the municipalities; which should enable municipalities to enhance the quality of their services, through greater knowledge about the service users, what affects their satisfaction and the degree of satisfaction with the provided services.

## Method

### Design

A partial explorative survey based study specifically designed in collaboration with users, the Norwegian Public Health Institute and researchers at the center of regional drug and alcohol competence center research department in the mid-region of Norway. The participating municipalities were selected by using a set of criteria to ensure a representative selection. The criteria were; municipalities from all the seven regions, number of inhabitants (small, medium and large municipalities from rural and urban areas) and KOSTRA (Municipality-State-Reporting) numbers[42]. Within each region a set of three municipalities were drawn on the basis of being a small municipality (4999 or fewer inhabitants), medium sized (5000–19,999 inhabitants) and large (over 20,000 inhabitants). The KOSTRA numbers are based on inhabitant figures in the respective municipalities and the economic parameters, such as the portion of personnel per year and portion of users of health services, which the municipalities operate within. The municipalities were randomly selected electronically on the basis of these criteria. A main selection of municipalities were drawn and invited to participate in the survey. In cases where one or more municipalities from the main sample could not participate, two similar municipality samples were randomly selected as replacements in order to maintain a representative selection of municipalities.

### Data collection

Data were collected in September and October 2017. The municipality coordinators were instructed to communicate the study rationale and aim of the study to the service owners. The participating services were selected by convenience since some service providers had other ongoing obligations at the time of recruitment. The users were involved in the design of the study by being a reference group for the project throughout the entire project period. Here they could bring in relevant questions to be included in the survey regarding satisfaction and other issues they had with the study. The survey was then tested among users in the relevant target group who filled it out and reported back to the principal investigator. A focus group with 4 users was established with the project group and the questionnaire items were discussed with the users resulting in minor revisions to the final questionnaire to improve face validity.

Services within the municipalities are referred to as primary services and include general practitioner, Norwegian Labour and Welfare Administration, drug counsellors, psychologist services, activity centres, low-threshold services, child support services, support/training contacts, and work /activity related services. If they are in need of more specialised services like detoxification or outpatient/inpatient services with more specialized personnel and medications they are referred by their primary doctor or social services for further assessment and treatment. The services had information about the survey and the survey itself was available in all waiting rooms. The questionnaire was handed out by secretaries to include as many as possible within the time set. The survey was handed out together with an envelope which also included an information and consent letter. The participants were instructed to fill out the form themselves, but if needed they could get help from personnel on site. An electronic survey was delivered to those who could not get to the centers, and a total of 19 users opted for this solution. All users in contact with the services provided at the time of recruitment were asked to fill out the survey. It was emphasized that participation in the survey was voluntary and that data would be kept anonymous. The inclusion criteria were that users had to be 18 or older have a present or previous substance use disorder and be recipients of one or more services from their municipality. A user is diagnosed with SUD or abuse according to the International Classification Diagnostic, version 10 which states that a SUD is a condition in which the use of one or more substances leads to clinically significant impairment or distress (ICD-10: WHO, 1993). An external company coordinated gathering and scanning of the questionnaires from the different service providers through an electronic solution. The completed questionnaires were sent by surface mail from the different service providers to the company which prepared the data for analysis.

The study protocol was reviewed and approved by the National Committee for Medical and Health Research Ethics (NEM) (application no. 2017/317).

### Sample size

According to national data in BrukerPlan 2016 [24], (National report from the SUD service providers) the estimated number of recipients of substance use services per 1000 inhabitants in Norway is 6.5 (highest in the smaller municipalities). By adding the numbers from the participating municipalities in this study, we can assume that the total number of inhabitants using SUD services is 5353. This means that the current sample constitutes a total of 9% of the total population receiving substance use services in the selected municipalities (491/5335). The range of municipalities selected might justify the representative selection of users, but since the selection of services within municipalities varied it is more questionable. However, to investigate what affects service user satisfaction might not create the same need for representability; the answers might be well provided by the ones included. Targeted sample size needed was 10 users per parameter in regression analysis which was achieved.

### Questionnaire and measurement instruments

The questionnaire included demographic items such as gender, age, education, and marital status. In addition, clinical items regarding alcohol consumption were included using the previously validated CAGE (Cutting down, Annoyance by criticism, Guilty feeling, and Eye-openers) [33] and the symptom Checklist-10 (SCL-10)[45]. CAGE is a measure of alcohol use and abuse. CAGE items have yes/no options and the cut-off point was set to 2 [13].

The SCL-10 is validated for general psychological distress and users are asked to rate their symptoms on the items during the last 7 days. A Global severity index (GSI) can be calculated from the SCL-10 by adding their score and dividing by the amount of items included in the scale to establish an overall indicator of symptom load. The GSI obtained Cronbach′s α of 0.907. A GSI score above 1.75 indicates that the patient is approaching a similar symptom load as patients with anxiety and mood disorders in general psychiatric health care [12]. SCL-10 is graded from “not bothered” to “considerably bothered”. The patients were asked to report on other clinical relevant variables; if they had any previous treatment for SUD disorder, age at onset of first use of alcohol intoxication, and whether they had used illicit drugs the last year (cannabis, amphetamines/methamphetamines, ecstasy/MDMA, cocaine, LSD, heroin, new synthetic drugs, illegal methadone or buprenorphine products). Items covering satisfaction were specifically designed to investigate what kind of services they had used, to what degree the services helped them in important life areas, whether they had received practical help and experiences with the personnel and overall service satisfaction. The 19 Satisfaction items were graded on a five-point Likert scale from “not at all” to “a large extent”.

### Statistical analyses

Mean distribution and percentages were used to present demographic and clinical variables. A principal components analysis (PCA) with iteration [21], varimax rotation and Kaizer’s criterion was performed on the satisfaction items. Cronbach’s alpha was used to test reliability of the indexes identified in PCA. Since some of the items lack responses from some users there is a difference of total n and n on some of the questionnaire items. For this reason n is provided for each question. In addition, the category «not relevant» was excluded from the analysis to give a percentage distribution of the satisfaction of those who have actually used the specific services in that particular item. Selection of variables was done a priori based on prior literature on personnel factors and mental health which has been found associated with satisfaction scores. For these reasons the selected variables were entered in the regression analysis together with demographics and clinical variables. Hierarchical block regression analysis was conducted to test the whether GSI, health status and municipality size explained variance in satisfaction scores, while adjusting for demographics and clinical variables. Demographic characteristics (age, gender, education and marital status) were entered in the first block as control variables. The second block consisted of the GSI scores and perceived physical health. In order to test whether clinical variables like alcohol abuse (CAGE cut-off), age at onset of first use of alcohol intoxication, or illicit use added to the explained variance above and beyond the demographics and clinical variables were entered in the third block. All of the above-mentioned analyses were conducted with IBM® SPSS® Statistics version 23.

## Results

### Sample characteristics

Out of the 491 included users, 70% were men. Mean age was 42 years (SD = 12.77, range = 18–75) with no significant gender differences. Single respondents (81%) constituted the majority of the sample, 5% were married, 12% living together with a partner and 2% widowed. High school was completed by 45%; college by 48 and 7% had a university degree. Unemployment was reported by 32, 48% were retired or on disability pension, 10% were in work training or students and 6% worked full time. Of the total sample 68% lived in their own house (owned or rented), 5% had no stable living arrangement, 3% were living with their partners with/without children or living with other family members/friends, 6% were in an institution and 4% lived in a hostel/hotel. Almost 50% had children and 9% of the total sample had daily care responsibility for their children.

### Clinical characteristics

**Fig. 1:**
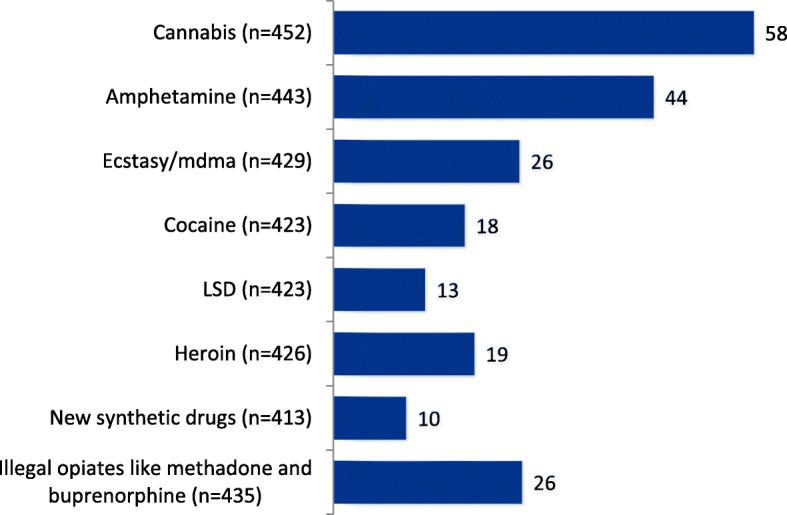
Use of illicit drugs last year. *Percentage display of users’ scores

**Fig. 2:**
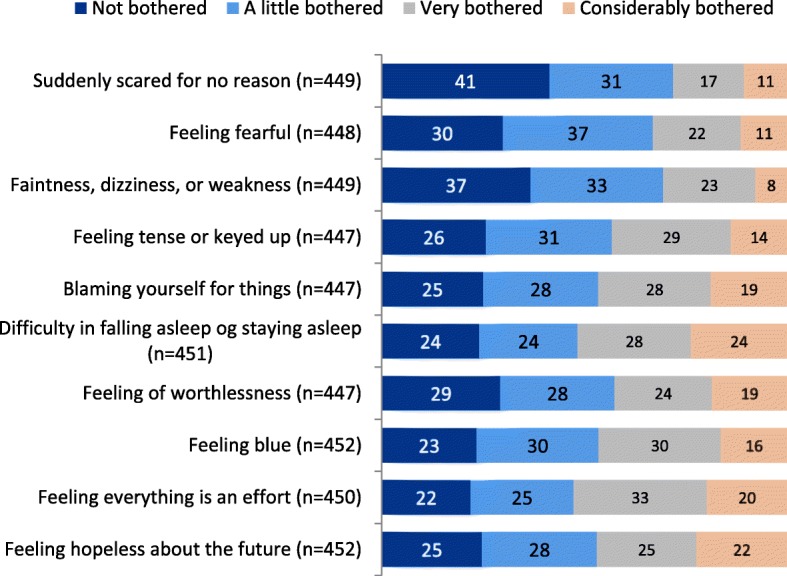
GSI symptom load among users of SUD services. *Percentage display of service users’ scores

A mixed patient group regarding substance use is represented in this study with 66% having used alcohol the last three months. Use of illicit drugs the last 12 months is displayed in Fig. 1. The most common illicit drug used was cannabis (58%) followed by amphetamines (44%). Perception of own health status showed that 22% viewed their physical health as poor, 30% fair, 29% good, 14% very good and 4% excellent. Regarding mental health, 23% reported that they assessed it to be poor, 31% fair, 30% good, 11% very good and 6% excellent. Mental health and symptom scores on single items representing the GSI are shown in Fig. 2. A total of 26% of 491 users had previously taken part in outpatient treatment for their SUD issues within the specialized health care system. Of the sample a total of 28% had received medically assisted substitution treatment for opioids and 16% had previously been admitted to a specialized inpatient clinic. A total of 40% (*n* = 446) had been in detoxification program 3 or more times and 31% had more than 3 previous inpatient admissions. The most frequently used health services were their general practitioner (78%), NAV (Norwegian Labour and Welfare Administration, 72%) and addiction counsellors (62%). In addition, 73% had been in a detoxification program at least once before and 67% had been in some kind of specialized SUD treatment prior to the use of services within the municipality.

Figure 2 displays scores on the SCL- 10 among the service users. On each item over half up to 78% reported that they to some extent or worse had experienced the listed symptoms during the last week. The Global Severity Index (GSI) showed that the mean was 2.29 for all ten items (SD = .769).

### Dimensionality of the satisfaction indexes

A Principal Components Analysis (PCA) was performed on the satisfaction items and it revealed three dimensions and the number of extracted dimensions were also confirmed using the scree plot (eigenvalues > 1). From this we computed three satisfaction indicators in addition to the GSI which were further used in regression analysis. The satisfaction indexes were overall satisfaction and important life areas (Cronbach’s α = .900), satisfaction with practical help (Cronbach’s α = .879), and satisfaction with personnel experiences (Cronbach’s α = .890). Additional file 1: Table S1a shows the items included in each dimension and Cronbach’s alpha for all dimensions. All reliability indices were satisfactory.

The first multiple regression with “overall satisfaction and important life areas” as dependent variable (DV) revealed that marital status, age and municipality size accounted for 7.7% (adjusted R^2^) of the total variance in the DV (Table 1). Age was significantly related to the satisfaction indicator as older service users were less satisfied than younger service users. This was also the case for marital status, as single respondents were less satisfied than service users with partners. Those in smaller municipalities were more satisfied than service users from larger municipalities. Introducing the GSI explained 11.9% (adjusted R^2^) of the variation in the DV and the change in R^2^ was significant. Those with higher scores on the GSI and high symptom pressure were less satisfied than respondents with lower scores. In the third and last block “illicit use” added significantly to the explained variance and the total model explained 15.6% of the variation (adjusted R^2^) in “overall satisfaction and important life areas”. Those with illicit use the last year were less satisfied with the services. Marital status did not exert any significant influences on the model in the last block.

**Table 1:** Regression analysis with “Overall satisfaction and important life areas” as dependent variable

Block	Indicators	*B*	CI 95%	F-change
1	Gender	.097	−.069;.459	4.621^b^
	Age	-.190^a^	−.024;.-.004	
	Education	.043	−.131;.259	
	Marital status	-.172^a^	−.711;-.093	
	Municipality size	-.190^a^	−.478;-.089	
2				6.131^a^
	Marital status	−.173 ^a^	−.706;-.102	
	Age	−.160 ^a^	−.021;-.002	
	Municipality size	−.176 ^a^	−.453;-.072	
	GSI	−.192 ^a^	−.388;-.068	
	Perceived physical health	.068	−.179;.060	
3				4.035^a^
	Marital status	−.160	−.673;-.077	
	Age	-.235^b^	−.027;-.007	
	Municipality size	-.131^a^	−.386;-.005	
	GSI	−.154 ^a^	−.342;-.023	
	CAGE Cut-off	.064	−.117;.351	
	Onset of first use	.113	−.006;.072	
	Illicit use last year	-.167^a^	−.603;-.059	

Dependent variable-overall satisfaction R^2^ = 0.194 CI 95% = Confidence interval, a *p* < 0.05; b *p* < 0.001

In the second regression model with the “satisfaction with practical help” as a dependent variable two out of three blocks were significant (Table 2). First block marital status as single was significantly associated with reduced satisfaction. Entering GSI in block two accounted for 3.6% of the variance in “satisfaction with practical help” (adjusted R^2^) together with marital status, and those with high GSI scores had lower satisfaction with practical help. In the last block onset of first intoxication of alcohol was a significant predictor of “satisfaction with practical help” and the older they are at first intoxication the more satisfied they are. The variables in the model explained a total of 5.4% of the variance (adjusted R^2^). When all variables were entered marital status was not a significant predictor in the overall model.

**Table 2:** Regression analysis with “Satisfaction with practical help” as dependent variable

Block	Indicators	*B*	CI 95%	F-change
1				1.931
	Gender	.104	−.021;.055	
	Age	−.018	−.012;.009	
	Education	.006	−.193;.215	
	Marital status	-.119^a^	−.704;-.014	
	Municipality size	−.057	−.289:.092	
2				4.560^a^
	Marital status	−.119 ^a^	−.710;-.027	
	GSI	-.174^a^	−.432;-.091	
	Perceived physical health	.040	−.081;.168	
3				2.941^a^
	GSI	−.145 ^a^	−.392;-.045	
	CAGE Cut-off	.000	−.258;.256	
	Onset of first intox of alcohol	.169^a^	.017;.087	
	Illicit use last year	−.006	−.312;.281	

Dependent variable- satisfaction practical help: R^2^ = .083 CI 95% = Confidence interval 95%, a p < 0.05; b p < 0.001

The third and last regression model with “Satisfaction with personnel experiences” as dependent variable showed that three out of three blocks were significant and in block one municipality size accounted for 2.5% (Adjusted R^2^) of the variation in “Satisfaction with personnel experiences” (Table 3). Those from larger municipalities were less satisfied. Introducing the GSI explains 9.5% (Adjusted R^2^) of the variance in the dependent variable and those with high GSI scores were less satisfied than those with lower symptom pressure. The last block “onset of first intoxication of alcohol” and “illicit use” adds to the explained variance in “Satisfaction with personnel experiences” and both were negatively associated with satisfaction. The total model explains 12.2% (Adjusted R^2^) of the variance in the dependent variable.

**Table 3:** Regression analysis with “Satisfaction with personnel experiences” as dependent variable

Block	Indicators	*B*	CI 95%	F-change
1				2.666^a^
	Gender	−.054	−.391;.134	
	Age	−.012	−.010;.008	
	Education	.031	−.133;.238	
	Marital status	−.056	−.456;.147	
	Municipality size	-.178^b^	−.447;-.109	
2				13.543 ^b^
	Municipality size	−.155	−.405;-.078	
	GSI	-.228^b^	−.488;-.170	
	Perceived physical health	−.056	−.172;.058	
3				4.238^b^
	Municipality size	−.130 ^a^	−.366;-.041	
	GSI	−.197 ^b^	−.434;-.121	
	CAGE Cut-off	.024	−.182;.287	
	Onset of first alcohol intoxication	.120 ^a^	.003;.066	
	Illicit use last year	-.137^a^	−.621;-.084	

Dependent variable-: Personnel experiences R^2^ = 0.149, CI 95% = Confidence interval 95%, a p < 0.05, b p < 0.001

### Satisfaction and single item scores

The single items belong to four categories covering overall satisfaction with services, satisfaction with practical help, satisfaction in important life areas and satisfaction with personnel experiences. Overall satisfaction shows that 54% (*n* = 452) to a large or great extent were satisfied with the services provided. More than half reported help when they need it (51%) and almost half that the services were available to them when needed (49%). Out of 407 service users, 41% were to a large or great extent satisfied with help to reduce or master their SUD issues. In terms of practical help, 76% (*n* = 242) reported that they were not at all or very little satisfied with help starting an education and 54% (*n* = 290) reported the same low satisfaction with help getting a job. Over half (52%; *n* = 368) reported that they were not at all or very little satisfied with help getting involved with meaningful daily activities. In terms of satisfaction on important life areas, 47% (*n* = 360) reported that they were not at all or very little satisfied with help with their economy, getting involved with exercise (58%; *n* = 374), and establishing a social network (62%; *n* = 361). Satisfaction with personnel experiences are somewhat higher than the previous ones and over half (56%; *n* = 434) noted that they to a large or great extent were satisfied that their needs were understood by the personnel. 58% (*n* = 433) reported that they to a large or great extent were satisfied with sufficient time and contact with personnel in charge and have trust in them (53%, *n* = 427). Also, 55% (*n* = 447) describe that they to a large or great extent were satisfied with how the personnel had treated them with respect and dignity. The remaining results are presented in Additional file 1: Table S1b.

## Discussion

This is on of the first study on satisfaction among service users of SUD services in the municipalities of Norway of this scale. To our knowledge, no other study in this vein has incorporated a relatively large random sample obtained from a representative set of municipalities across any country. In order to investigate factors that differentiate satisfaction among users of SUD services within the municipalities, demographic and clinical data have been controlled for.

The service users represent a mixed patient group in terms of types of substance used with a majority of males, single individuals, and users who are unemployed or on disability pension. The most used substances were alcohol, cannabis and amphetamines. The demographics are similar to previous studies conducted in the specialized health care and municipality sectors in Norway [2, 24, 32, 44]. They received, to a large extent, services from their general practitioner, Norwegian Labor and Welfare Administration and addiction counsellors.

A PCA revealed three satisfaction indexes, “Overall satisfaction and important life areas”, “Satisfaction with practical help” and “Satisfaction with personnel experience”. The first regression analysis on “Overall satisfaction and important life areas” which included physical and mental issues, establishing a social network and getting started with physical activity showed that age was negatively related to satisfaction, and the effect was moderate. Older users report less satisfaction than younger respondents do. This result contradicts studies which have shown that older respondents tend to be more satisfied [5]. One reason can be that previous studies have investigated populations within specialized systems and psychiatric inpatient facilities and not municipality services. Another potential reason might be that older users with a SUD have a better understanding of their needs and a stronger opinion about whether or not the services can meet these needs properly. Moret et al. [30] suggested a non-linear influence of patient age on satisfaction with hospital care. In the present study we have service user up to the age of 75, and this might have influenced our results. In addition, this age association we see in our study might also be affected by the severity of the users within this sample who are more severely affected by their SUD shown by multiple treatments within the specialised health care system prior to the use of services within the municipalities. Age or other demographics were not associated with the two other satisfaction measures when controlling for the remaining variables in the model.

### Satisfaction and municipality size

Analysis of “Overall satisfaction and important life areas” and “Satisfaction with personnel experience” revealed that municipality size was negatively associated with satisfaction. It seems like users in the smaller municipalities are more satisfied than those living in larger ones. This might be due to the transparency in smaller ones, which may have a more clear-cut available services and closer follow-up of the service users. This can be more difficult to achieve in larger municipalities, where a higher number of users and more pressure on the services may reduce both the availability and possibilities of adequate follow-up of the users. The difficulties smaller municipalities have in recruiting competent personnel may reduce satisfaction, but in our study, we see greater satisfaction within smaller municipalities. Urban and larger municipalities might have easier access to high competent personnel, but might experience more turnover than smaller municipalities with less services and personnel in competition. It might be that smaller municipalities have longer lasting relations between personnel and users that increase trust, understanding of needs, and cooperation. The size of the municipalities seems to affect overall satisfaction, satisfaction with important life areas and personnel experiences, but the effect is small, and we have limited knowledge about why these differences appear and need more research to investigate this further.

GSI scores were negatively associated with all three satisfaction indexes, which support prior studies showing that anxiety, distress and depression may be strong predictors of patient satisfaction [5]. Results should be interpreted with caution considering the effect of the association was small in the “Overall satisfaction and important life areas” and “Satisfaction with practical help” and moderate in “Satisfaction with personnel experiences”. High psychological stress may affect the view and satisfaction with services given because of the nature of the disorder, or it can reflect the fact that their needs in this area are not met like previous research found in terms of health related quality of life [43]. Here especially men reported that their needs in terms of psychological services were not met by the service providers and patients’ psychological health affected their health-related quality of life. Increasing services to stabilize symptom pressure and more personnel with psychiatric competence may improve satisfaction in this area.

In addition to GSI, “Onset of first alcohol intoxication” was positively associated with “Satisfaction with practical help” and “Satisfaction with personnel experiences”, however the effect was small in both. The older the users were when they had their first intoxication from alcohol, the more satisfied they seem to be with the help they have received. The reason for this may be that those with early alcohol intoxication have a more complex display of SUD, and that it is more difficult to fulfill their needs. Further, illicit substance use was negatively associated with satisfaction. Those who had used such substances the last year were less satisfied with the health services. Illicit use may affect and increase mental disorders and vice versa, like amphetamine increases the risk of psychotic symptoms and more needs in terms of factors such as social network and economy that make it harder to meet all of their needs [7]. Illicit use also increases needs in terms of social network, economy, etc., which in turn makes it harder to meet all the user’s needs.

The results on degree of satisfaction on single item scores suggest that more than half of the current sample were satisfied with the overall services provided by their municipalities. In terms of practical help the users were most satisfied with help getting a residence, and less satisfied with help with living in a residency, get work/education, economic help and getting started with activities they find meaningful. Regarding satisfaction in important life areas, including their physical and mental health, the users were most satisfied with help with their SUD problems and related coping, but less satisfied with help to deal with physical and mental issues, establishing a social network and getting started with physical activity. The areas showing less satisfaction, like social network and dealing with physical and mental issues, were related to relapse and are important elements found to improve to increase positive treatment outcome in previous research [4, 25, 38, 41].

Studies have shown that social support through one’s network is important in order to change behavior in a successful manner, like reducing or quitting substance use [20, 34].The social network is also important to remain abstinent and this network should encourage healthy activities and different strategies to deal with life events [10]. Despite the fact that users may have complicated or nonexistent social networks, it is still an important area to work on. A small focus group study we executed on patients in an inpatient setting in mid-region of Norway stressed this point. Even though the patients found it a difficult and somewhat daunting task to work on, they also considered it a highly important part to work on in treatment for their long term recovery [36].

Social network and engaging in daily activities like work and physical exercise has also shown to be important in keeping patients with co-occurring mental and SUD issues abstinent [37].Considering daily activities like work obligations, Finn et al. [15]found that having responsibilities the next day strongly affects decisions on attending treatment and amount of drinking, which underlines the importance of social inclusion through having a job [15]. The focus and responsibilities in Norwegian SUD services are based on a biopsychosocial approach, which makes it important to also include specific services for this group targeting work and education. These areas are considered highly important to reduce or avoid substances altogether. These services are preferably executed with personnel with SUD competence or in cooperation with personnel with this competence so that the services can target specific needs. The services for work and education should enhance understanding of the disorder and to a greater degree target specific needs in terms of SUD and employment so they get the same opportunities like any other unemployed seeker. The low satisfaction with these areas in this study might suggest that the services are not targeting these needs properly and enhancing understanding and specify services might improve satisfaction.

Service users in our study showed that their satisfaction is affected by personnel experience and that the service users were most satisfied with the information provided and being met with respect and understanding. Trust in personnel’s competence has been associated with higher satisfaction in other studies [5] and the service users report high satisfaction in this area. In the current study, more than half reported that they get enough time with their counsellors and trust in their ability to assist them. The importance of personnel competence and importance is documented in previous studies [3, 35]. There could be closer connections between users and personnel in smaller municipalities, securing the alliance between them, and perhaps more stability among the personnel which in turn leads to the higher satisfaction scores. Less pressure in terms of numbers of users might provide personnel in smaller municipalities with more time to attend each user’s needs. However, this should be subjected to further research.

### Limitations

The use of a self-report instrument has some limitations in terms of response bias, social desirability bias and validity of the questions. A majority of the respondents reported high overall satisfaction, but we do believe that the range of items in the survey and responses to these has ensured a reduction in these biases. The satisfaction scores also resemble scores from other national treatment studies and our study supports those found previous study by [19]. In addition, a representative sample from randomly drawn municipalities also strengthens our results. The effect sizes are, however, small to moderate and the results should be interpreted with caution. The need for a broad approach when investigating what is associated with satisfaction among users of SUD services within the municipalities warrants the questions in our survey. Some of them are taken from satisfaction surveys previously executed on inpatient patients in specialized service, but some were added based on focus group executed together with the users who stated their importance to measure their satisfaction. However, it is still a limitation that this designed survey has not been tested for its validity and reliability previously, although part of it is based on well-known satisfaction surveys [19, 27]. If we only wanted to investigate users’ degree of satisfaction it would have been more problematic. However, we asked them multiple questions, and the questions were not divided into the sections revealed in PCA, to investigate what we should be including in the future when investigating the degree of satisfaction. The variables found to be strongly associated with satisfaction should be included to reveal the true satisfaction experienced by the users to increase the correspondence between actual and self-reported satisfaction.

### Conclusion

Satisfaction with SUD services within the municipalities was negatively associated with age, GSI, municipality size, onset of first alcohol intoxication and illicit use last year. Age-targeted services to meet the demands of older users may increase satisfaction and lead to better treatment outcomes. Also, targeting specific needs according to severity, duration of use, type of substance and psychiatric symptom pressure is of great importance. Finally, the size of the municipality is associated with satisfaction and should be examined further to investigate explanatory factors for higher satisfaction within smaller compared to larger municipality service providers. Overall satisfaction among users of SUD services within different municipalities in Norway show that a majority is satisfied with the services provided and that they receive these when needed. However, there are several areas that need to be improved to meet user needs, increase satisfaction and improve the outcome of these services. Interventions to improve physical and mental health are of great importance, as is helping users to establish a social network, start an education, getting a job, and economic help. These areas give day-to-day life meaning and purpose which can compete with substance use. We do believe that the results found in this study are of use beyond our borders to countries with similar provision of services. A majority of western countries have now the same understanding of treating addiction with multiple services to reduce substance use and reintegrate users back to society and increase satisfaction with these services might in turn improve the users outcome and reintegration.

## Additional file

Additional file 1: **Table S1**a. Dimensionality and reliability of the satisfaction of services instrument. b. Single item satisfaction scores covering overall satisfaction, satisfaction with practical help, satisfaction with important life areas and personnel experiences. (DOCX 22 kb)

## Abbreviations

CAGE: Cutting down, Annoyance by criticism, Guilty feeling, and Eye-openers
DV: Dependent variable
GSI: Global severity index
KOSTRA: Municipality-State-Reporting
NAV: Norwegian Labour and Welfare Administration
NEM: The National Committee for Medical and Health Research Ethics
PCA: Principal Components Analysis
SCL-10: The Symptom Checklist-10
SUD: Substance use disorder
