# Supplementary material for: Assessing health service satisfaction among users with substance use disorders within the municipalities in Norway
Source: Subst Abuse Treat Prev Policy. 2019 May 6;14:18. doi: 10.1186/s13011-019-0207-4 (PMC6501295; doi:10.1186/s13011-019-0207-4)
Supplement: Supplementary file 1 — Table S1a. Dimensionality and reliability of the satisfaction of services instrument. b. Single item satisfaction scores covering overall satisfaction, satisfaction with practical help, satisfaction with important life areas and personnel experiences. (DOCX 22 kb) [file 13011_2019_207_MOESM1_ESM.docx]

Additional file 1

Material section

Table S1a: Dimensionality and reliability of the satisfaction of services instrument

| Item | Dim 1  Overall satisfaction and vital areas | Dim 2  Satisfaction Practical help | Dim 3  Satisfaction Personnel experiences |
| --- | --- | --- | --- |
| Overall are the services you have received from the municipality satisfactory? | **,691** | - | ,450 |
| Have the help you have received helped you in your everyday living? | **,700** | - | ,405 |
| To what extent do you experience help when you need it? | **,691** | - | ,507 |
| Is the availability of the services satisfying? | **,640** | - | ,484 |
| To what extend have you experienced satisfactory help with.. |  |  |  |
| …your mental health | **,626** | - | - |
| …with your physical health or medical disease | **,606** | - | - |
| …to start exercising | **,614** | ,517 | - |
| …to establish a social network | **,591** | ,519 | - |
| …to reduce/master your substance use problems | **,554** | - | - |
| To what extent have you experienced practical help from services to… |  |  |  |
| …get a place to live | - | **,679** | - |
| …to master your daily living in the home | - | **,739** | - |
| …get a job | - | **,785** | - |
| …starting a education | - | **,836** | - |
| ….master your economy | - | **,653** | - |
| …start with meaningful daily activities | ,560 | **,561** | - |
| Satisfaction personnel experience |  |  |  |
| The personnel in charge has understood your needs | - | - | **,788** |
| Have experienced enough time and contact with the personnel in charge | - | - | **,763** |
| Have trust in the personnel in charge | - | - | **,829** |
| Met with dignity and respect by personnel | - | - | **,748** |
| Cronbach’s α | 0.900 | 0.879 | 0.890 |

Factor loading <0.40

### Table S1b: Single item satisfaction scores covering overall satisfaction, satisfaction with practical help, satisfaction with important life areas and personnel experiences

| Item | N | Not at all  (%) | Low extent(%) | Somewhat  (%) | To a large extent(%) | To a great extent(%) |
| --- | --- | --- | --- | --- | --- | --- |
| Overall are the services you have received from the municipality satisfactory? | 452 | 5 | 13 | 29 | 35 | 19 |
| Have the help you have received helped you in your everyday living? | 453 | 11 | 15 | 32 | 26 | 16 |
| To what extent do you experience help when you need it? | 453 | 8 | 17 | 25 | 34 | 17 |
| Is the availability of the services satisfying? | 451 | 6 | 16 | 29 | 35 | 14 |
| To what extent have you experienced practical help from services to… |  |  |  |  |  |  |
| …get a place to live | 359 | 26 | 14 | 17 | 20 | 23 |
| …to master your daily living in the home | 347 | 25 | 18 | 27 | 17 | 13 |
| …get a job | 290 | 36 | 18 | 23 | 12 | 11 |
| …starting an education | 242 | 57 | 19 | 12 | 5 | 7 |
| ….master your economy | 360 | 30 | 17 | 26 | 13 | 14 |
| …start with meaningful daily activities | 368 | 33 | 19 | 24 | 15 | 9 |
| To what extend have you experienced satisfactory help with.. |  |  |  |  |  |  |
| …your Mental Health | 406 | 21 | 19 | 25 | 21 | 13 |
| …with your physical health or medical disease | 396 | 20 | 19 | 27 | 23 | 11 |
| …to start exercising | 374 | 33 | 25 | 20 | 14 | 9 |
| …to establish a social network | 361 | 39 | 23 | 22 | 10 | 5 |
| …to reduce/master your substance use problems | 407 | 21 | 12 | 26 | 24 | 17 |
| The personnel in charge has understood your needs | 434 | 9 | 12 | 23 | 29 | 27 |
| Have experienced enough time and contact with the personnel in charge | 433 | 9 | 14 | 19 | 34 | 24 |
| Have trust in the personnel in charge | 427 | 8 | 8 | 20 | 29 | 34 |
| Met with dignity and respect by personnel | 447 | 4 | 13 | 28 | 42 | 13 |
